# Supplementary material for: On the enigma of Palaenigma wrangeli (Schmidt), a conulariid with a partly non-mineralized skeleton
Source: PeerJ. 2021 Nov 2;9:e12374. doi: 10.7717/peerj.12374 (PMC8570166; doi:10.7717/peerj.12374)
Supplement: Supplemental Information 4 — Specimen GIT 655-1 from Kükita 24 drillcore, south of Mustvee, north-east Estonia, Vormsi Stage, and specimen GIT 655-2, from Ellavere drillcore, Järva County, north-east Estonia, Nabala Stage. [file peerj-09-12374-s004.docx]

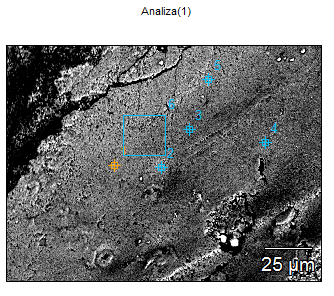


Image Name: Adapical, distal part of the pillar

(GIT 655-2)

Image Resolution: 2048 by 1536

Image Pixel Size: 0.08 µm

Acc. Voltage: 15.0 kV

Magnification: 1200

Detector: ULTRADRY


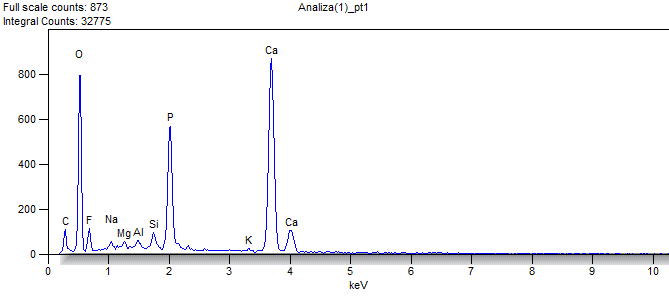


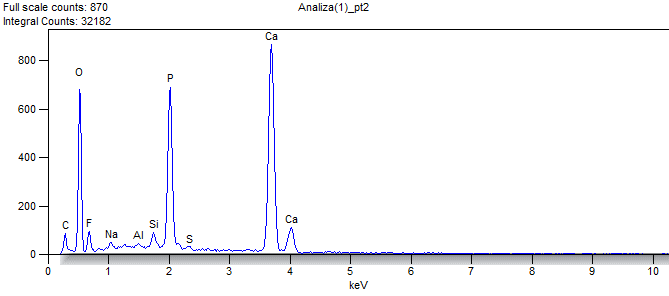


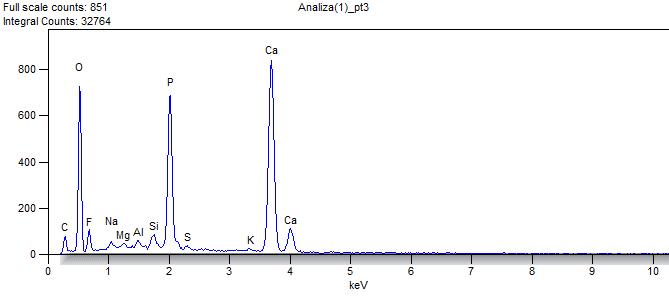


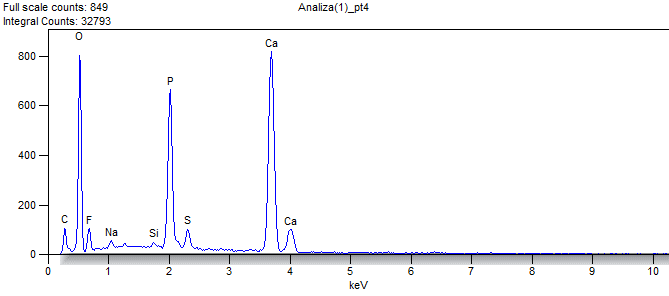


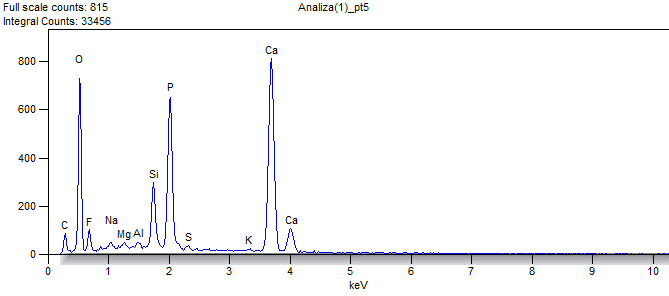


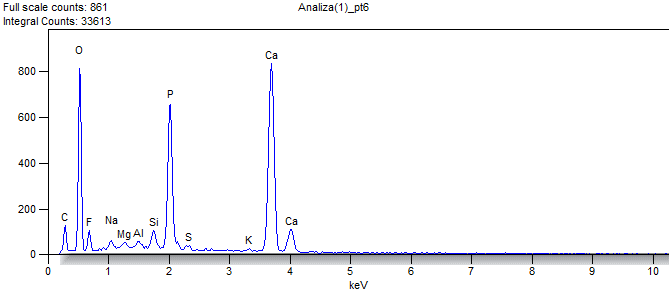


Weight %

|  | ***O*** | ***F*** | ***Na*** | ***Mg*** | ***Al*** | ***Si*** | ***P*** | ***S*** | ***K*** | ***Ca*** |
| --- | --- | --- | --- | --- | --- | --- | --- | --- | --- | --- |
| ***Analiza(1)_pt1*** | 43.017 | 5.707 | 0.690 | 0.492 | 0.480 | 1.263 | 11.023 |  | 0.315 | 37.014 |
| ***Analiza(1)_pt2*** | 39.502 | 5.085 | 0.719 |  | 0.214 | 1.072 | 13.471 | 0.324 |  | 39.612 |
| ***Analiza(1)_pt3*** | 40.424 | 5.395 | 0.670 | 0.317 | 0.397 | 1.135 | 13.235 | 0.323 | 0.381 | 37.724 |
| ***Analiza(1)_pt4*** | 42.522 | 5.522 | 0.780 |  |  | 0.352 | 12.290 | 1.685 |  | 36.849 |
| ***Analiza(1)_pt5*** | 39.522 | 5.277 | 0.543 | 0.389 | 0.293 | 4.334 | 13.138 | 0.271 | 0.248 | 35.984 |
| ***Analiza(1)_pt6*** | 41.891 | 4.998 | 0.880 | 0.495 | 0.399 | 1.292 | 12.457 | 0.442 | 0.424 | 36.722 |

Atom %

|  | ***O*** | ***F*** | ***Na*** | ***Mg*** | ***Al*** | ***Si*** | ***P*** | ***S*** | ***K*** | ***Ca*** |
| --- | --- | --- | --- | --- | --- | --- | --- | --- | --- | --- |
| ***Analiza(1)_pt1*** | 61.252 | 6.844 | 0.684 | 0.461 | 0.405 | 1.025 | 8.108 |  | 0.183 | 21.039 |
| ***Analiza(1)_pt2*** | 58.129 | 6.301 | 0.736 |  | 0.187 | 0.899 | 10.240 | 0.238 |  | 23.269 |
| ***Analiza(1)_pt3*** | 58.810 | 6.610 | 0.678 | 0.304 | 0.342 | 0.940 | 9.946 | 0.234 | 0.227 | 21.908 |
| ***Analiza(1)_pt4*** | 60.907 | 6.661 | 0.778 |  |  | 0.287 | 9.094 | 1.204 |  | 21.069 |
| ***Analiza(1)_pt5*** | 57.587 | 6.475 | 0.551 | 0.373 | 0.253 | 3.597 | 9.888 | 0.197 | 0.148 | 20.930 |
| ***Analiza(1)_pt6*** | 60.276 | 6.056 | 0.882 | 0.469 | 0.340 | 1.059 | 9.259 | 0.318 | 0.250 | 21.093 |


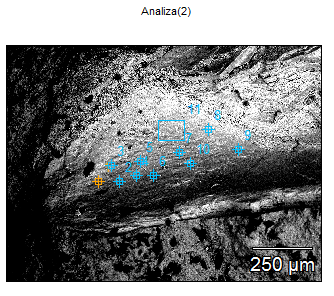


Image Name: Adapical, distal part of the pillar

(GIT 655-2)

Image Resolution: 2048 by 1536

Image Pixel Size: 0.65 µm

Acc. Voltage: 15.0 kV

Magnification: 150

Detector: ULTRADRY


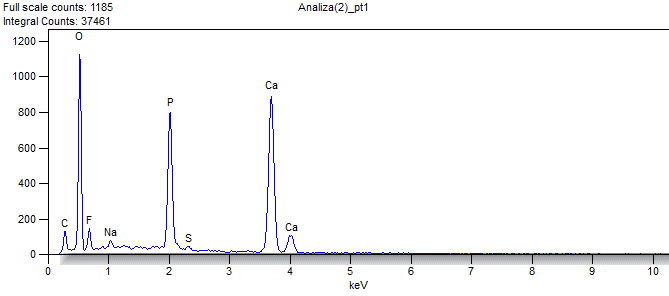


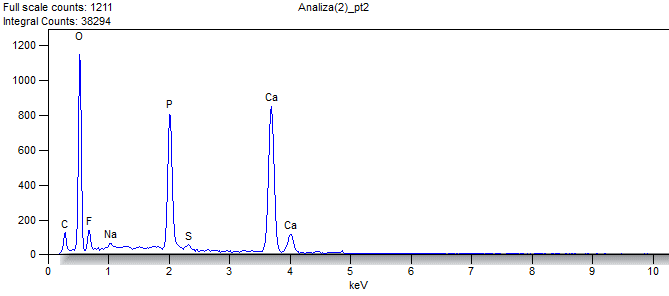


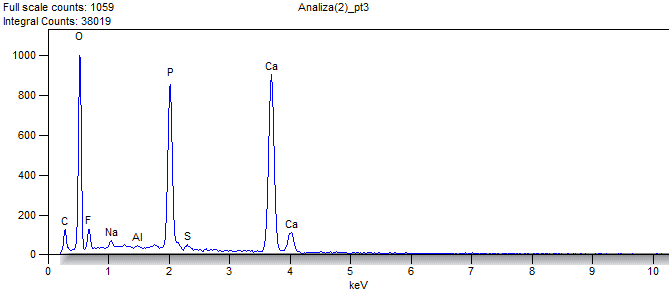


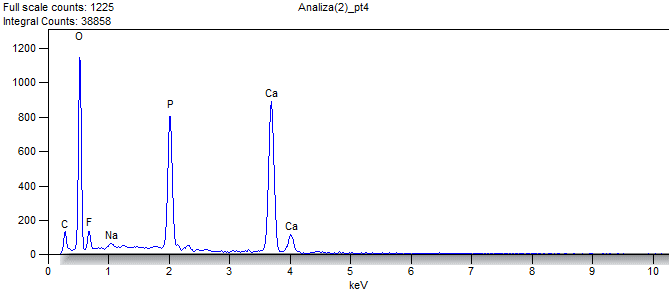


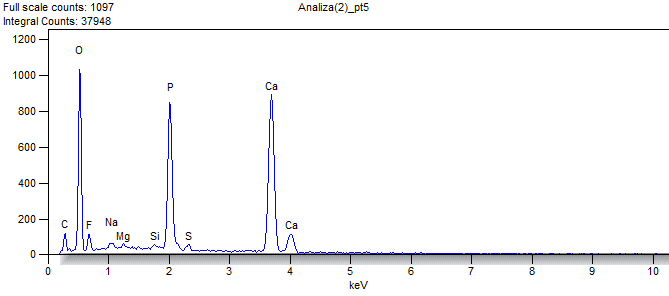


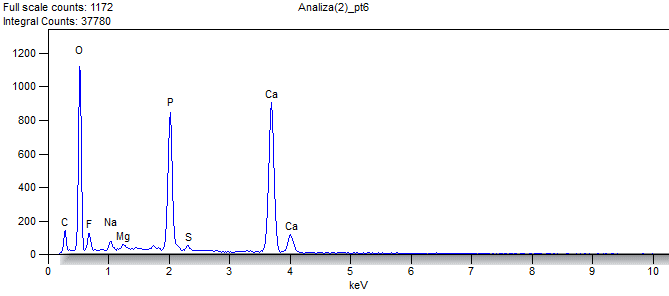


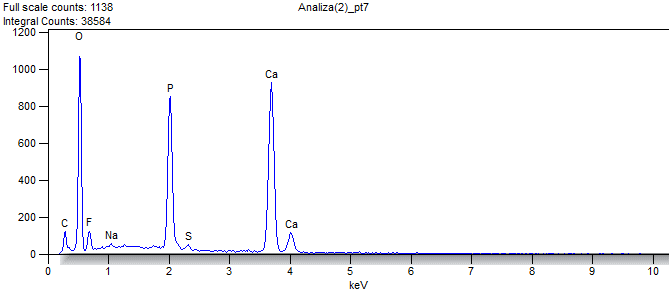


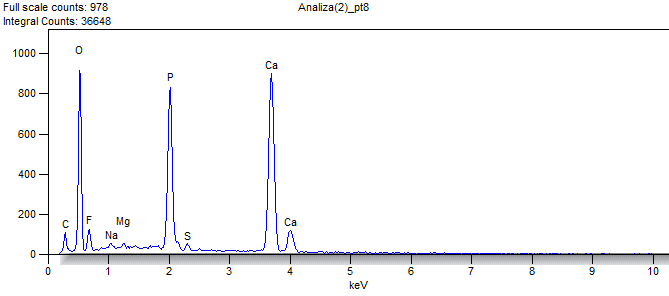


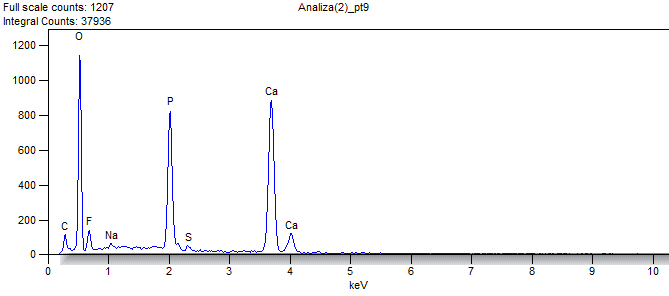


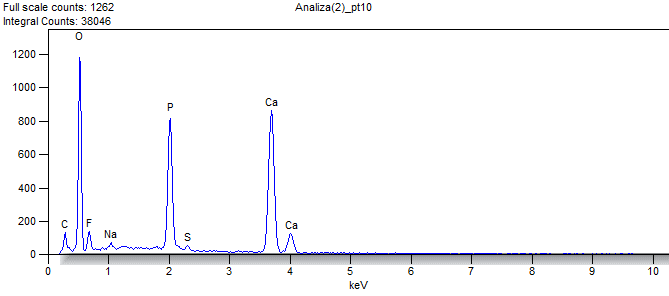


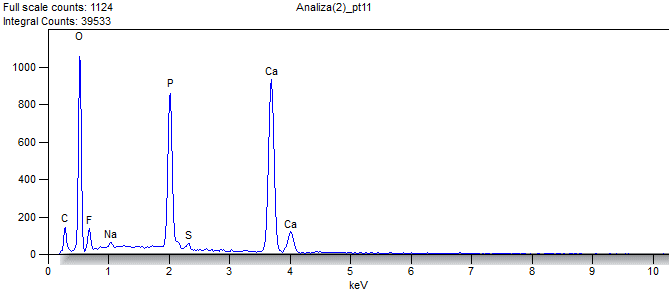


Weight %

|  | ***O*** | ***F*** | ***Na*** | ***Mg*** | ***Al*** | ***Si*** | ***P*** | ***S*** | ***Ca*** | ***Ba*** |
| --- | --- | --- | --- | --- | --- | --- | --- | --- | --- | --- |
| ***Analiza(2)_pt1*** | 46.406 | 5.594 | 0.797 |  |  |  | 12.982 | 0.456 | 33.764 |  |
| ***Analiza(2)_pt2*** | 45.804 | 5.698 | 0.754 |  |  |  | 13.264 | 0.735 | 32.207 | 1.538 |
| ***Analiza(2)_pt3*** | 44.366 | 5.346 | 0.641 |  | 0.075 |  | 14.394 | 0.443 | 34.736 |  |
| ***Analiza(2)_pt4*** | 47.355 | 5.036 | 0.768 |  |  |  | 13.836 |  | 33.004 |  |
| ***Analiza(2)_pt5*** | 45.147 | 4.251 | 0.757 | 0.247 |  | 0.253 | 13.968 | 0.688 | 34.690 |  |
| ***Analiza(2)_pt6*** | 46.008 | 5.424 | 0.912 | 0.350 |  |  | 13.679 | 0.597 | 33.030 |  |
| ***Analiza(2)_pt7*** | 45.660 | 5.739 | 0.467 |  |  |  | 13.835 | 0.610 | 33.689 |  |
| ***Analiza(2)_pt8*** | 43.149 | 6.043 | 0.439 | 0.290 |  |  | 14.396 | 0.536 | 35.146 |  |
| ***Analiza(2)_pt9*** | 45.203 | 6.360 | 0.550 |  |  |  | 13.106 | 0.504 | 33.054 | 1.223 |
| ***Analiza(2)_pt10*** | 46.878 | 6.074 | 0.665 |  |  |  | 13.104 | 0.593 | 32.686 |  |
| ***Analiza(2)_pt11*** | 44.731 | 5.988 | 0.528 |  |  |  | 13.468 | 0.658 | 34.626 |  |

Atom %

|  | ***O*** | ***F*** | ***Na*** | ***Mg*** | ***Al*** | ***Si*** | ***P*** | ***S*** | ***Ca*** | ***Ba*** |
| --- | --- | --- | --- | --- | --- | --- | --- | --- | --- | --- |
| ***Analiza(2)_pt1*** | 64.378 | 6.535 | 0.770 |  |  |  | 9.303 | 0.316 | 18.698 |  |
| ***Analiza(2)_pt2*** | 64.168 | 6.722 | 0.735 |  |  |  | 9.598 | 0.514 | 18.011 | 0.251 |
| ***Analiza(2)_pt3*** | 62.593 | 6.352 | 0.629 |  | 0.063 |  | 10.490 | 0.312 | 19.563 |  |
| ***Analiza(2)_pt4*** | 65.360 | 5.854 | 0.738 |  |  |  | 9.865 |  | 18.184 |  |
| ***Analiza(2)_pt5*** | 63.617 | 5.045 | 0.742 | 0.229 |  | 0.203 | 10.167 | 0.484 | 19.513 |  |
| ***Analiza(2)_pt6*** | 63.909 | 6.345 | 0.881 | 0.320 |  |  | 9.815 | 0.414 | 18.315 |  |
| ***Analiza(2)_pt7*** | 63.667 | 6.739 | 0.453 |  |  |  | 9.965 | 0.425 | 18.752 |  |
| ***Analiza(2)_pt8*** | 61.232 | 7.222 | 0.434 | 0.271 |  |  | 10.553 | 0.379 | 19.909 |  |
| ***Analiza(2)_pt9*** | 63.398 | 7.512 | 0.537 |  |  |  | 9.495 | 0.353 | 18.506 | 0.200 |
| ***Analiza(2)_pt10*** | 64.598 | 7.049 | 0.638 |  |  |  | 9.328 | 0.408 | 17.980 |  |
| ***Analiza(2)_pt11*** | 62.781 | 7.078 | 0.516 |  |  |  | 9.764 | 0.461 | 19.400 |  |


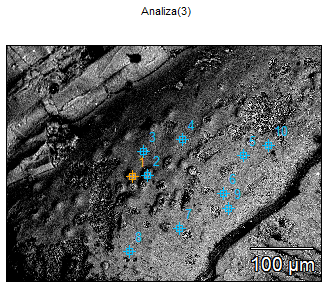


Image Name: area with papillae in a distal,

adapical lpart of the pillar

(GIT 655-2)

Image Resolution: 2048 by 1536

Image Pixel Size: 0.25 µm

Acc. Voltage: 15.0 kV

Magnification: 400

Detector: ULTRADRY


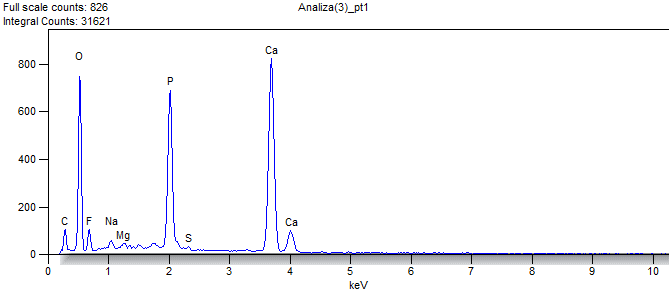


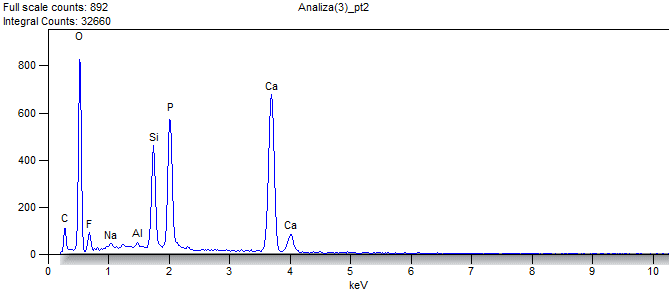


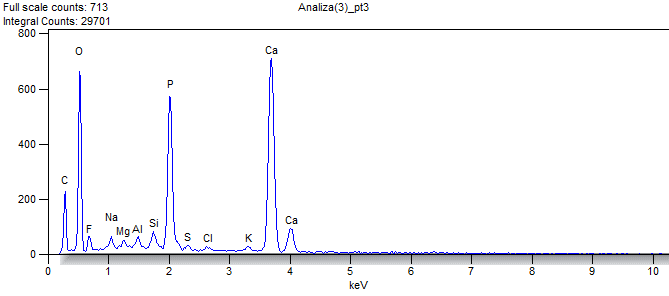


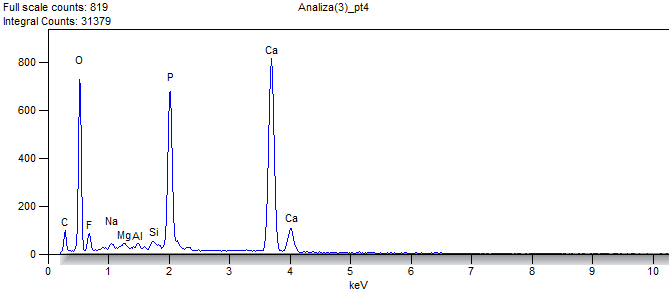


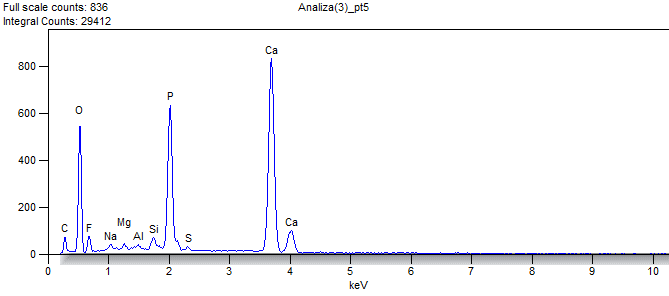


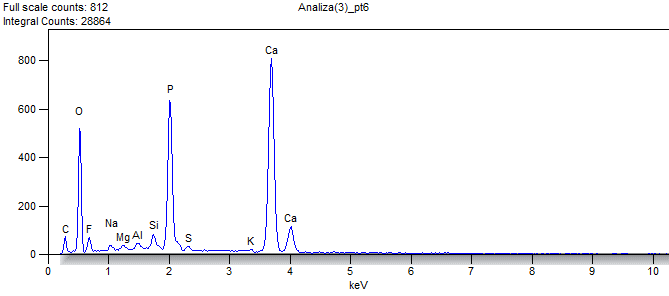


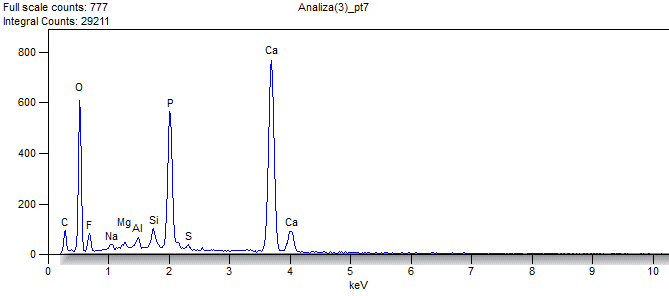


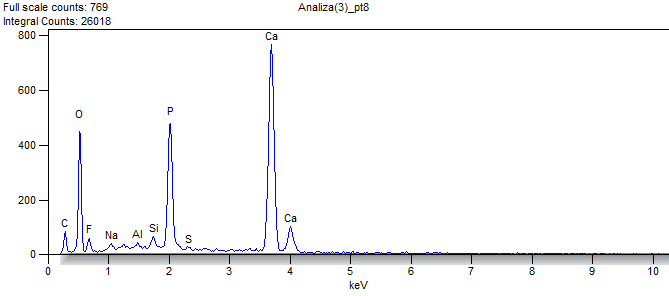


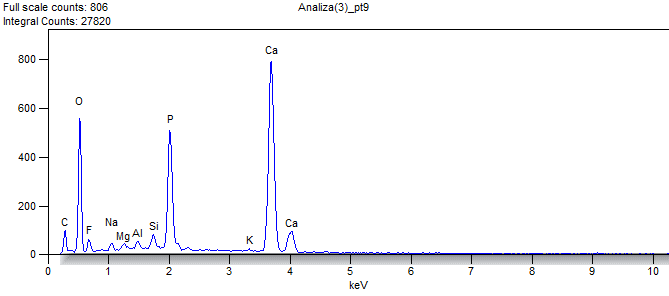


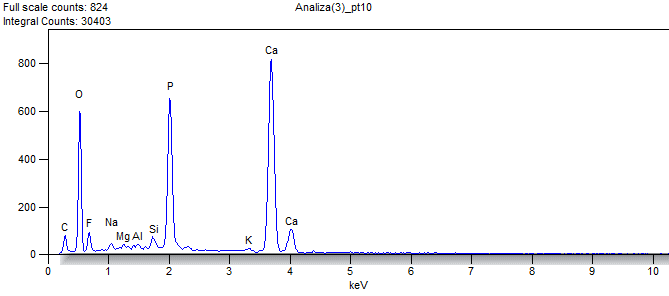


Weight %

|  | ***O*** | ***F*** | ***Na*** | ***Mg*** | ***Al*** | ***Si*** | ***P*** | ***S*** | ***Cl*** | ***K*** | ***Ca*** |
| --- | --- | --- | --- | --- | --- | --- | --- | --- | --- | --- | --- |
| ***Analiza(3)_pt1*** | 42.074 | 5.155 | 0.781 | 0.470 |  |  | 13.859 | 0.236 |  |  | 37.426 |
| ***Analiza(3)_pt2*** | 42.742 | 4.667 | 0.573 |  | 0.278 | 7.649 | 12.170 |  |  |  | 31.921 |
| ***Analiza(3)_pt3*** | 40.672 | 4.134 | 0.997 | 0.527 | 0.631 | 1.283 | 12.998 | 0.400 | 0.425 | 0.530 | 37.403 |
| ***Analiza(3)_pt4*** | 41.674 | 5.085 | 0.348 | 0.371 | 0.190 | 0.598 | 13.707 |  |  |  | 38.027 |
| ***Analiza(3)_pt5*** | 37.269 | 4.737 | 0.589 | 0.370 | 0.184 | 1.031 | 13.829 | 0.330 |  |  | 41.659 |
| ***Analiza(3)_pt6*** | 35.990 | 3.989 | 0.581 | 0.384 | 0.344 | 1.203 | 14.572 | 0.369 |  | 0.370 | 42.198 |
| ***Analiza(3)_pt7*** | 38.897 | 5.332 | 0.507 | 0.452 | 0.688 | 1.590 | 12.726 | 0.363 |  |  | 39.445 |
| ***Analiza(3)_pt8*** | 35.726 | 5.078 | 0.734 |  | 0.371 | 0.998 | 12.637 | 0.211 |  |  | 44.245 |
| ***Analiza(3)_pt9*** | 38.504 | 4.237 | 0.717 | 0.464 | 0.485 | 1.247 | 12.042 |  |  | 0.379 | 41.924 |
| ***Analiza(3)_pt10*** | 37.967 | 5.543 | 0.581 |  |  | 0.755 | 13.904 |  |  | 0.562 | 40.688 |

Atom %

|  | ***O*** | ***F*** | ***Na*** | ***Mg*** | ***Al*** | ***Si*** | ***P*** | ***S*** | ***Cl*** | ***K*** | ***Ca*** |
| --- | --- | --- | --- | --- | --- | --- | --- | --- | --- | --- | --- |
| ***Analiza(3)_pt1*** | 60.552 | 6.247 | 0.782 | 0.445 |  |  | 10.303 | 0.170 |  |  | 21.501 |
| ***Analiza(3)_pt2*** | 60.522 | 5.566 | 0.565 |  | 0.233 | 6.170 | 8.902 |  |  |  | 18.043 |
| ***Analiza(3)_pt3*** | 59.329 | 5.078 | 1.012 | 0.507 | 0.546 | 1.066 | 9.794 | 0.291 | 0.280 | 0.316 | 21.780 |
| ***Analiza(3)_pt4*** | 60.260 | 6.192 | 0.351 | 0.353 | 0.163 | 0.493 | 10.238 |  |  |  | 21.950 |
| ***Analiza(3)_pt5*** | 56.004 | 5.995 | 0.616 | 0.366 | 0.164 | 0.883 | 10.734 | 0.248 |  |  | 24.990 |
| ***Analiza(3)_pt6*** | 54.860 | 5.120 | 0.616 | 0.385 | 0.311 | 1.045 | 11.474 | 0.281 |  | 0.231 | 25.677 |
| ***Analiza(3)_pt7*** | 57.326 | 6.618 | 0.520 | 0.438 | 0.601 | 1.335 | 9.688 | 0.267 |  |  | 23.206 |
| ***Analiza(3)_pt8*** | 54.464 | 6.519 | 0.778 |  | 0.335 | 0.867 | 9.951 | 0.160 |  |  | 26.925 |
| ***Analiza(3)_pt9*** | 57.481 | 5.327 | 0.745 | 0.456 | 0.429 | 1.061 | 9.286 |  |  | 0.232 | 24.983 |
| ***Analiza(3)_pt10*** | 56.563 | 6.955 | 0.602 |  |  | 0.641 | 10.700 |  |  | 0.342 | 24.197 |


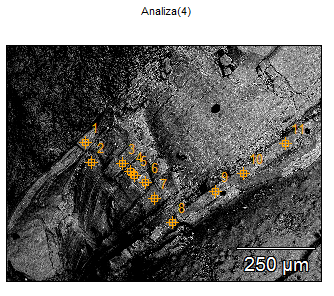


Image Name: layering of the pillar in a distal,

adapical part

(GIT 655-2)

Image Resolution: 2048 by 1536

Image Pixel Size: 0.49 µm

Acc. Voltage: 15.0 kV

Magnification: 200

Detector: ULTRADRY


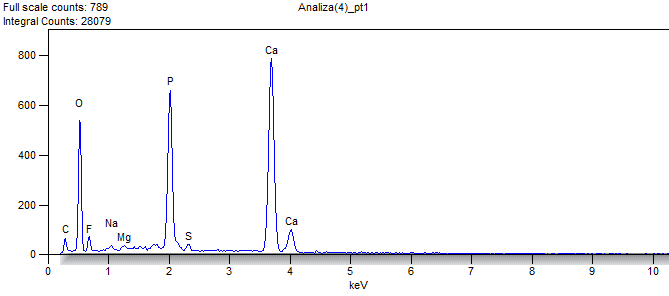


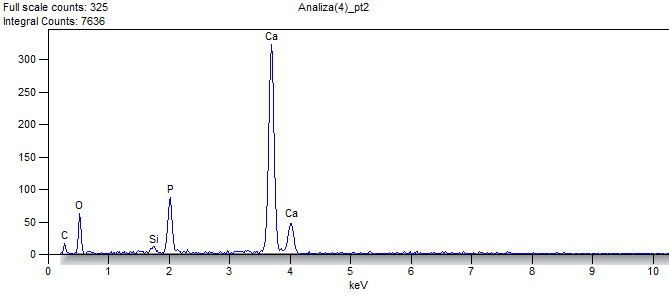


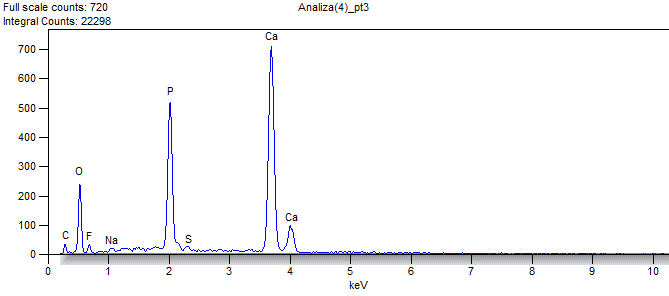


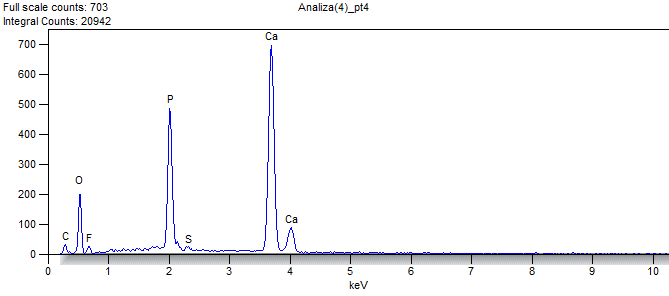


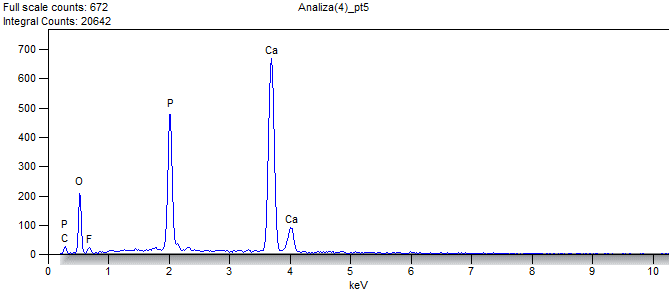


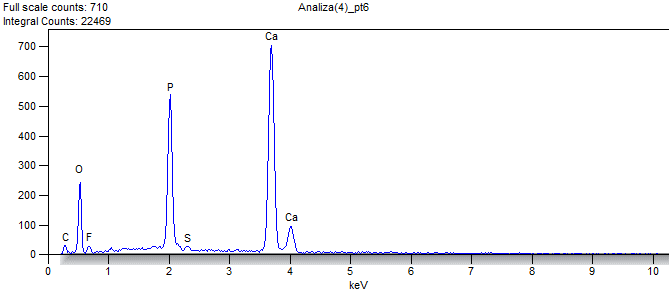


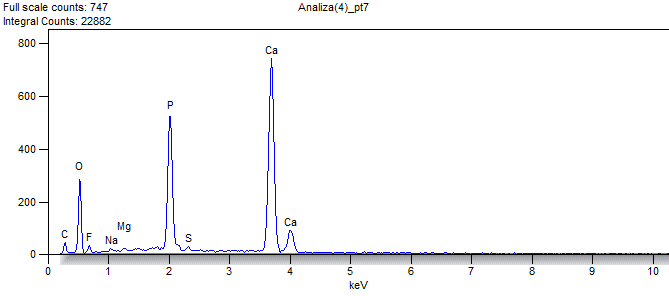


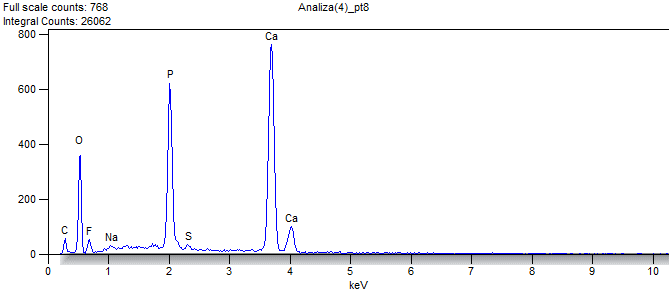


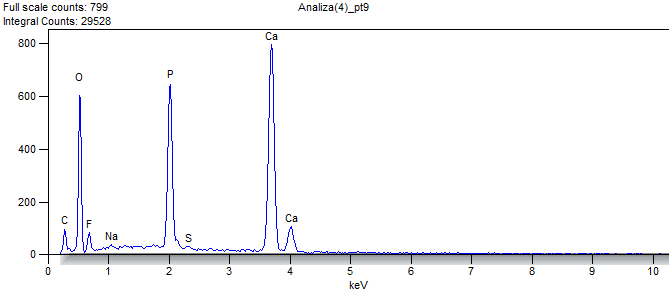


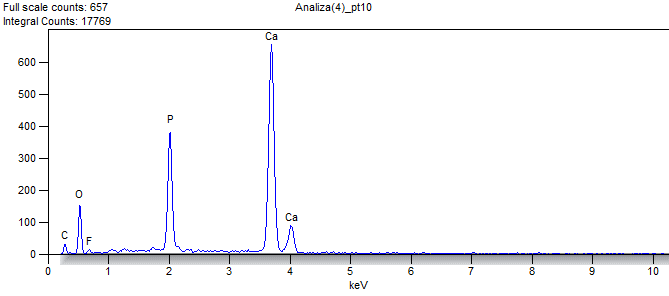


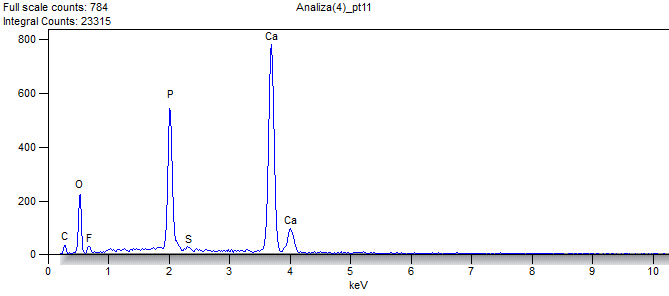


Weight %

|  | ***O*** | ***F*** | ***Na*** | ***Mg*** | ***Si*** | ***P*** | ***S*** | ***Ca*** |
| --- | --- | --- | --- | --- | --- | --- | --- | --- |
| ***Analiza(4)_pt1*** | 37.691 | 4.151 | 0.508 | 0.306 |  | 15.305 | 0.596 | 41.441 |
| ***Analiza(4)_pt2*** | 22.000 |  |  |  | 0.877 | 8.336 |  | 68.787 |
| ***Analiza(4)_pt3*** | 28.184 | 2.887 | 0.362 |  |  | 16.154 | 0.406 | 52.006 |
| ***Analiza(4)_pt4*** | 26.870 | 2.823 |  |  |  | 15.976 | 0.423 | 53.908 |
| ***Analiza(4)_pt5*** | 26.091 | 2.496 |  |  |  | 16.245 |  | 55.169 |
| ***Analiza(4)_pt6*** | 28.501 | 2.781 |  |  |  | 16.632 | 0.512 | 51.573 |
| ***Analiza(4)_pt7*** | 30.171 | 2.794 | 0.430 | 0.288 |  | 16.182 | 0.546 | 49.589 |
| ***Analiza(4)_pt8*** | 31.379 | 4.968 | 0.524 |  |  | 16.015 | 0.579 | 46.535 |
| ***Analiza(4)_pt9*** | 38.564 | 5.288 | 0.450 |  |  | 14.312 | 0.352 | 41.033 |
| ***Analiza(4)_pt10*** | 24.221 | 1.149 |  |  |  | 14.970 |  | 59.661 |
| ***Analiza(4)_pt11*** | 26.204 | 3.172 |  |  |  | 16.491 | 0.373 | 53.759 |

Atom %

|  | ***O*** | ***F*** | ***Na*** | ***Mg*** | ***Si*** | ***P*** | ***S*** | ***Ca*** |
| --- | --- | --- | --- | --- | --- | --- | --- | --- |
| ***Analiza(4)_pt1*** | 56.688 | 5.258 | 0.532 | 0.303 |  | 11.891 | 0.447 | 24.880 |
| ***Analiza(4)_pt2*** | 40.543 |  |  |  | 0.920 | 7.935 |  | 50.602 |
| ***Analiza(4)_pt3*** | 46.837 | 4.040 | 0.419 |  |  | 13.867 | 0.337 | 34.500 |
| ***Analiza(4)_pt4*** | 45.366 | 4.014 |  |  |  | 13.933 | 0.356 | 36.332 |
| ***Analiza(4)_pt5*** | 44.518 | 3.587 |  |  |  | 14.318 |  | 37.577 |
| ***Analiza(4)_pt6*** | 47.283 | 3.886 |  |  |  | 14.253 | 0.424 | 34.154 |
| ***Analiza(4)_pt7*** | 49.107 | 3.830 | 0.487 | 0.309 |  | 13.605 | 0.444 | 32.219 |
| ***Analiza(4)_pt8*** | 49.757 | 6.634 | 0.579 |  |  | 13.117 | 0.458 | 29.456 |
| ***Analiza(4)_pt9*** | 57.319 | 6.619 | 0.466 |  |  | 10.988 | 0.261 | 24.346 |
| ***Analiza(4)_pt10*** | 42.690 | 1.705 |  |  |  | 13.629 |  | 41.976 |
| ***Analiza(4)_pt11*** | 44.383 | 4.525 |  |  |  | 14.428 | 0.316 | 36.347 |


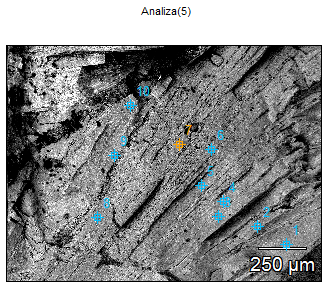


Image Name: Layers building the pillar below the

adapicalmost part

(GIT 655-2)

Image Resolution: 2048 by 1536

Image Pixel Size: 0.82 µm

Acc. Voltage: 15.0 kV

Magnification: 120

Detector: ULTRADRY


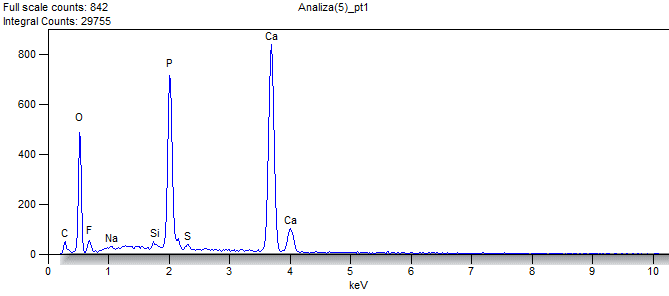


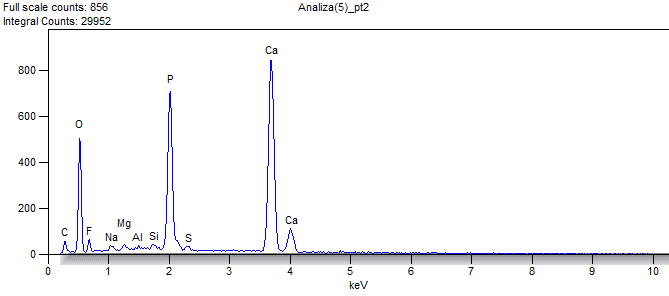


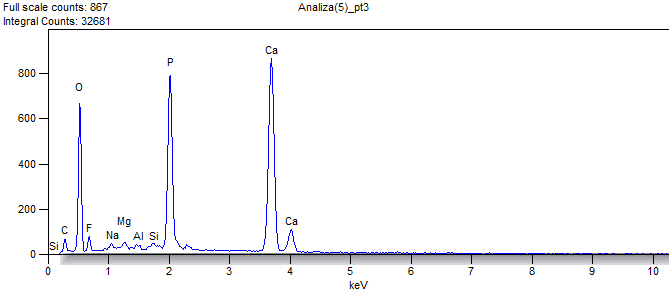


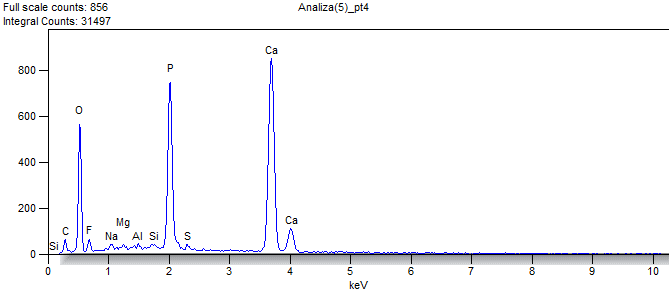


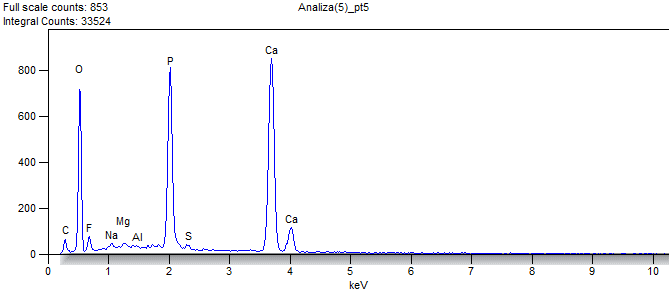


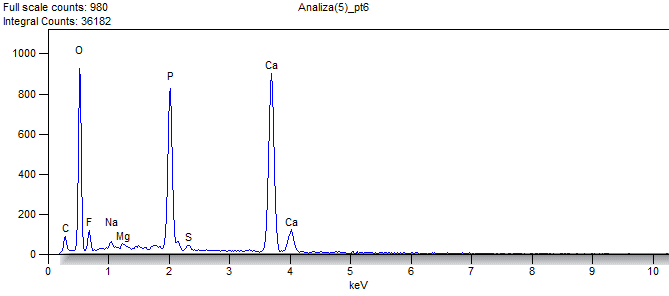


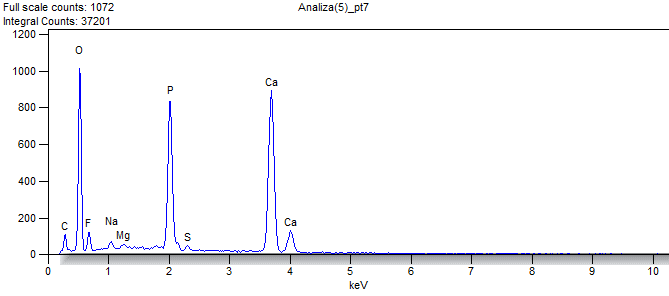


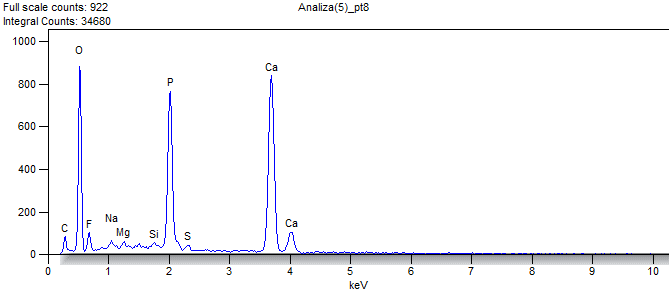


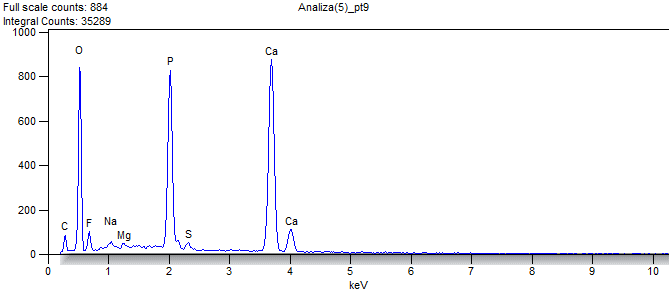


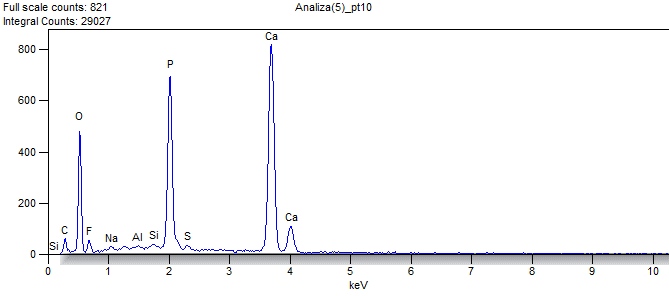


Weight %

|  | ***O*** | ***F*** | ***Na*** | ***Mg*** | ***Al*** | ***Si*** | ***P*** | ***S*** | ***Ca*** |
| --- | --- | --- | --- | --- | --- | --- | --- | --- | --- |
| ***Analiza(5)_pt1*** | 37.018 | 5.007 | 0.330 |  |  | 0.453 | 15.422 | 0.475 | 41.295 |
| ***Analiza(5)_pt2*** | 36.509 | 3.850 | 0.611 | 0.433 | 0.019 | 0.335 | 15.578 | 0.445 | 42.221 |
| ***Analiza(5)_pt3*** | 40.798 | 3.747 | 0.502 | 0.456 | 0.113 | 0.389 | 15.591 |  | 38.403 |
| ***Analiza(5)_pt4*** | 38.497 | 3.794 | 0.490 | 0.218 | 0.044 | 0.281 | 15.094 | 0.420 | 41.162 |
| ***Analiza(5)_pt5*** | 42.084 | 4.164 | 0.466 | 0.213 | 0.000 |  | 15.245 | 0.423 | 37.405 |
| ***Analiza(5)_pt6*** | 44.659 | 5.121 | 0.687 | 0.362 |  |  | 14.090 | 0.480 | 34.600 |
| ***Analiza(5)_pt7*** | 46.119 | 5.214 | 0.688 | 0.256 |  |  | 13.650 | 0.424 | 33.649 |
| ***Analiza(5)_pt8*** | 44.716 | 4.724 | 0.724 | 0.431 |  | 0.346 | 13.666 | 0.449 | 34.944 |
| ***Analiza(5)_pt9*** | 42.984 | 5.335 | 0.695 | 0.281 |  |  | 14.593 | 0.648 | 35.464 |
| ***Analiza(5)_pt10*** | 36.453 | 3.877 | 0.436 |  | 0.118 | 0.413 | 15.501 | 0.524 | 42.680 |

Atom %

|  | ***O*** | ***F*** | ***Na*** | ***Mg*** | ***Al*** | ***Si*** | ***P*** | ***S*** | ***Ca*** |
| --- | --- | --- | --- | --- | --- | --- | --- | --- | --- |
| ***Analiza(5)_pt1*** | 55.741 | 6.350 | 0.345 |  |  | 0.389 | 11.996 | 0.357 | 24.822 |
| ***Analiza(5)_pt2*** | 55.497 | 4.928 | 0.646 | 0.433 | 0.017 | 0.290 | 12.232 | 0.337 | 25.620 |
| ***Analiza(5)_pt3*** | 59.755 | 4.622 | 0.511 | 0.440 | 0.098 | 0.325 | 11.796 |  | 22.453 |
| ***Analiza(5)_pt4*** | 57.630 | 4.783 | 0.510 | 0.215 | 0.039 | 0.240 | 11.672 | 0.314 | 24.598 |
| ***Analiza(5)_pt5*** | 60.927 | 5.077 | 0.469 | 0.203 | 0.000 |  | 11.401 | 0.306 | 21.617 |
| ***Analiza(5)_pt6*** | 62.884 | 6.073 | 0.673 | 0.336 |  |  | 10.248 | 0.337 | 19.448 |
| ***Analiza(5)_pt7*** | 64.187 | 6.111 | 0.666 | 0.234 |  |  | 9.813 | 0.294 | 18.694 |
| ***Analiza(5)_pt8*** | 63.059 | 5.611 | 0.710 | 0.400 |  | 0.278 | 9.955 | 0.316 | 19.671 |
| ***Analiza(5)_pt9*** | 61.263 | 6.403 | 0.690 | 0.263 |  |  | 10.743 | 0.461 | 20.177 |
| ***Analiza(5)_pt10*** | 55.542 | 4.974 | 0.462 |  | 0.107 | 0.358 | 12.200 | 0.398 | 25.959 |


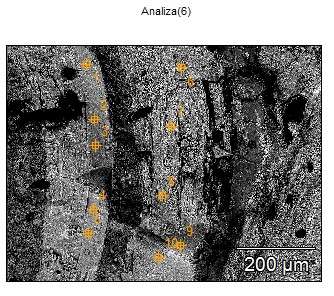


Image Name: diaphragms connecting the pillars

(GIT 655-2)

Image Resolution: 2048 by 1536

Image Pixel Size: 0.39 µm

Acc. Voltage: 15.0 kV

Magnification: 250

Detector: ULTRADRY


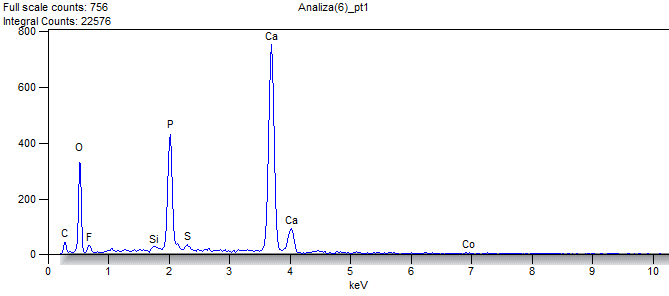


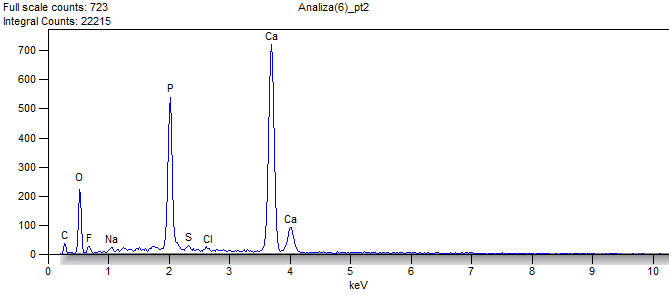


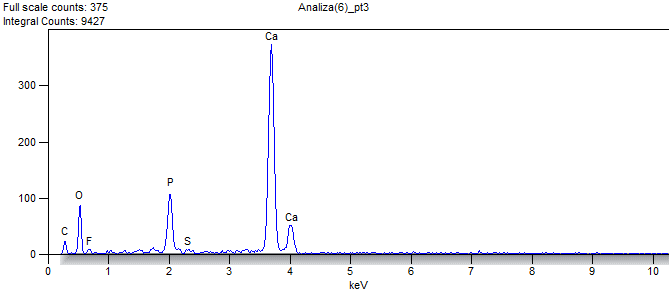


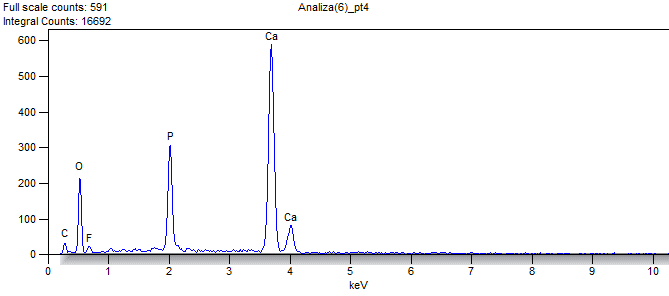


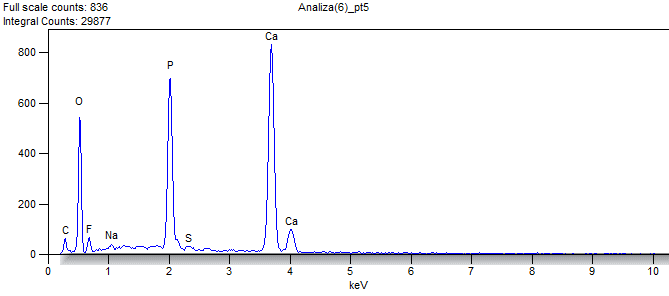


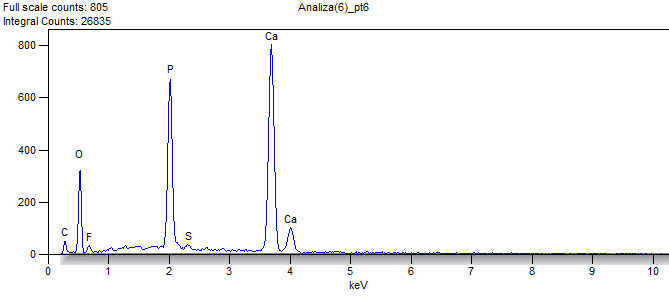


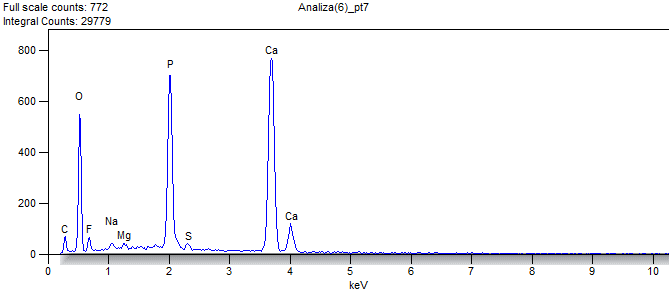


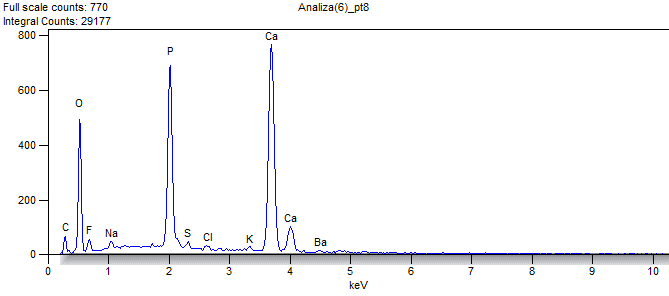


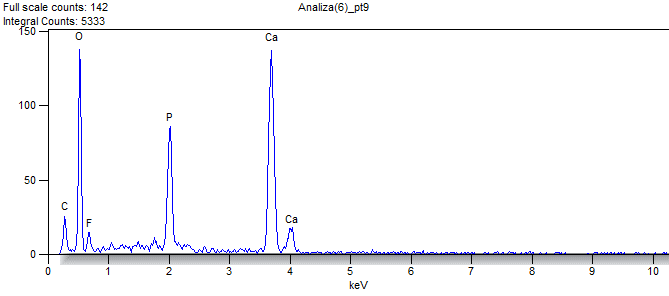


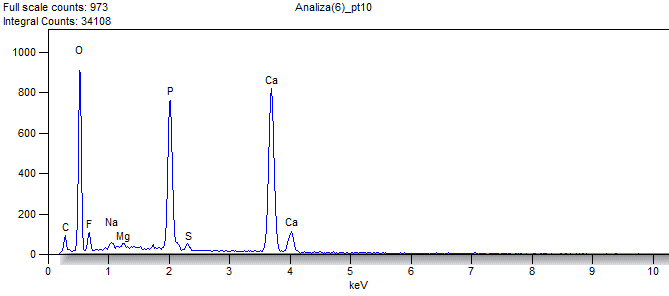


Weight %

|  | ***O*** | ***F*** | ***Na*** | ***Mg*** | ***Si*** | ***P*** | ***S*** | ***Cl*** | ***K*** | ***Ca*** | ***Co*** | ***Ba*** |
| --- | --- | --- | --- | --- | --- | --- | --- | --- | --- | --- | --- | --- |
| ***Analiza(6)_pt1*** | 33.103 | 3.288 |  |  | 0.328 | 12.338 | 0.790 |  |  | 48.458 | 1.696 |  |
| ***Analiza(6)_pt2*** | 27.432 | 2.562 | 0.417 |  |  | 16.381 | 0.463 | 0.428 |  | 52.318 |  |  |
| ***Analiza(6)_pt3*** | 26.459 | 1.923 |  |  |  | 8.614 | 0.449 |  |  | 62.556 |  |  |
| ***Analiza(6)_pt4*** | 32.934 | 3.243 |  |  |  | 11.653 |  |  |  | 52.171 |  |  |
| ***Analiza(6)_pt5*** | 38.207 | 3.967 | 0.523 |  |  | 15.562 | 0.454 |  |  | 41.288 |  |  |
| ***Analiza(6)_pt6*** | 30.851 | 3.067 |  |  |  | 17.645 | 0.705 |  |  | 47.732 |  |  |
| ***Analiza(6)_pt7*** | 37.226 | 4.394 | 0.650 | 0.318 |  | 15.615 | 0.526 |  |  | 41.272 |  |  |
| ***Analiza(6)_pt8*** | 36.042 | 4.182 | 0.703 |  |  | 15.499 | 0.565 | 0.558 | 0.425 | 40.060 |  | 1.966 |
| ***Analiza(6)_pt9*** | 45.811 | 6.693 |  |  |  | 10.696 |  |  |  | 36.800 |  |  |
| ***Analiza(6)_pt10*** | 45.680 | 5.329 | 0.653 | 0.236 |  | 14.161 | 0.549 |  |  | 33.392 |  |  |

Atom %

|  | ***O*** | ***F*** | ***Na*** | ***Mg*** | ***Si*** | ***P*** | ***S*** | ***Cl*** | ***K*** | ***Ca*** | ***Co*** | ***Ba*** |
| --- | --- | --- | --- | --- | --- | --- | --- | --- | --- | --- | --- | --- |
| ***Analiza(6)_pt1*** | 52.854 | 4.421 |  |  | 0.299 | 10.176 | 0.630 |  |  | 30.886 | 0.735 |  |
| ***Analiza(6)_pt2*** | 45.988 | 3.617 | 0.486 |  |  | 14.186 | 0.387 | 0.324 |  | 35.012 |  |  |
| ***Analiza(6)_pt3*** | 45.838 | 2.805 |  |  |  | 7.708 | 0.388 |  |  | 43.261 |  |  |
| ***Analiza(6)_pt4*** | 52.686 | 4.368 |  |  |  | 9.629 |  |  |  | 33.316 |  |  |
| ***Analiza(6)_pt5*** | 57.318 | 5.012 | 0.546 |  |  | 12.059 | 0.340 |  |  | 24.726 |  |  |
| ***Analiza(6)_pt6*** | 49.797 | 4.169 |  |  |  | 14.711 | 0.568 |  |  | 30.755 |  |  |
| ***Analiza(6)_pt7*** | 56.071 | 5.573 | 0.681 | 0.315 |  | 12.149 | 0.396 |  |  | 24.815 |  |  |
| ***Analiza(6)_pt8*** | 55.460 | 5.419 | 0.753 |  |  | 12.319 | 0.434 | 0.388 | 0.267 | 24.607 |  | 0.352 |
| ***Analiza(6)_pt9*** | 63.926 | 7.865 |  |  |  | 7.709 |  |  |  | 20.499 |  |  |
| ***Analiza(6)_pt10*** | 63.713 | 6.260 | 0.634 | 0.217 |  | 10.203 | 0.382 |  |  | 18.592 |  |  |


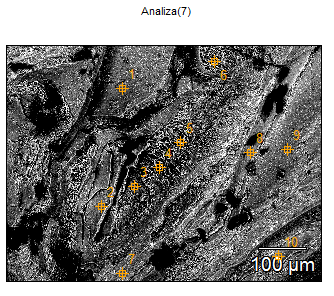


Image Name: microbial filaments and surrounded skeletal layers

(GIT 655-2)

Image Resolution: 2048 by 1536

Image Pixel Size: 0.33 µm

Acc. Voltage: 15.0 kV

Magnification: 300

Detector: ULTRADRY


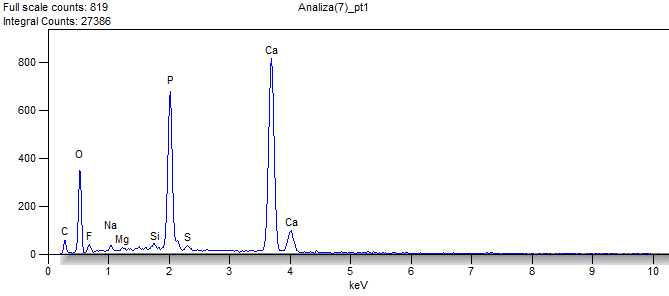


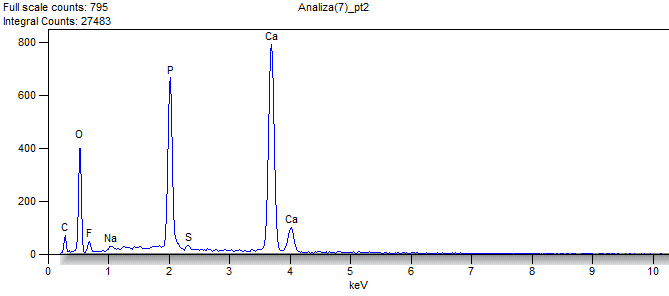


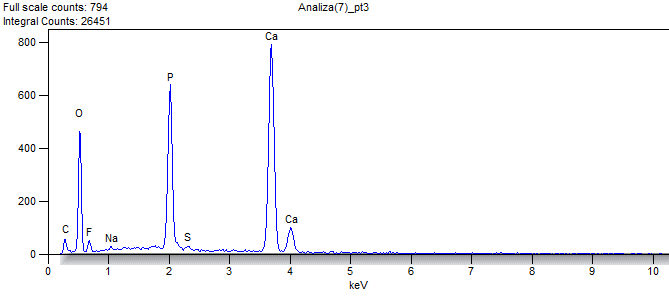


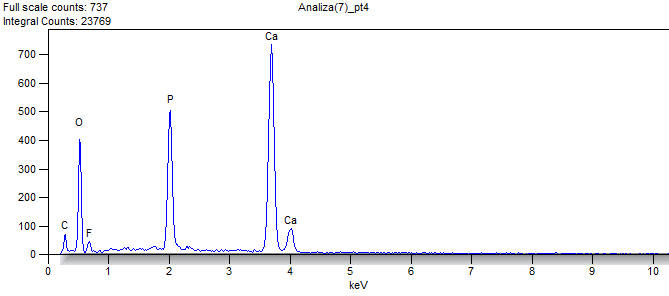


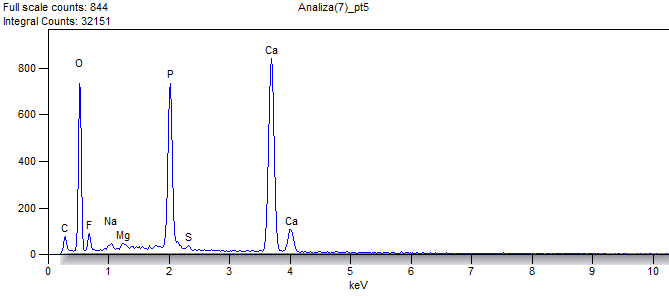


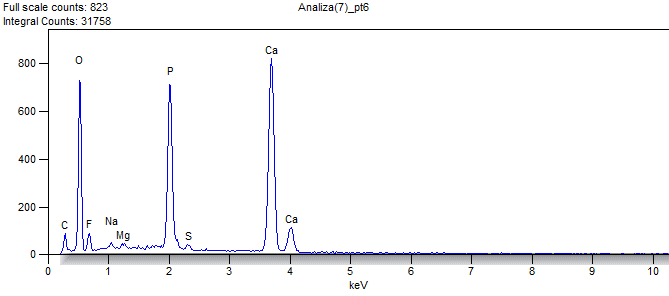


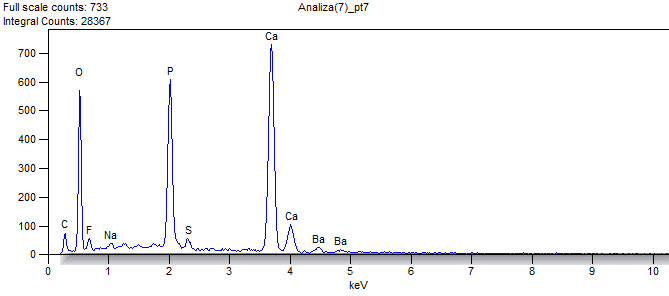


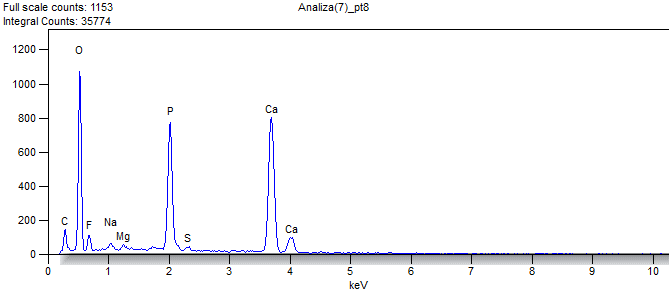


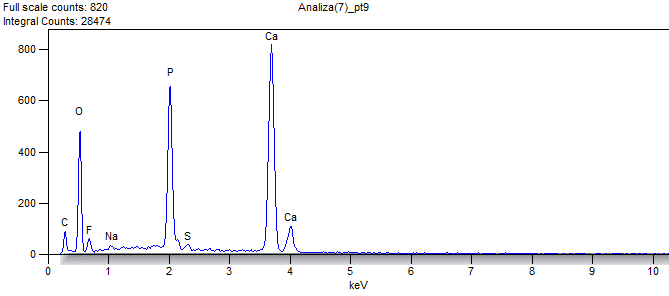


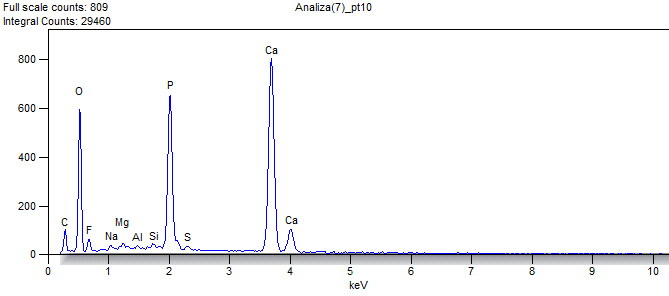


Weight %

|  | ***O*** | ***F*** | ***Na*** | ***Mg*** | ***Al*** | ***Si*** | ***P*** | ***S*** | ***Ca*** | ***Ba*** |
| --- | --- | --- | --- | --- | --- | --- | --- | --- | --- | --- |
| ***Analiza(7)_pt1*** | 32.522 | 3.191 | 0.655 | 0.296 |  | 0.336 | 16.569 | 0.470 | 45.960 |  |
| ***Analiza(7)_pt2*** | 33.972 | 4.033 | 0.520 |  |  |  | 16.062 | 0.509 | 44.904 |  |
| ***Analiza(7)_pt3*** | 37.523 | 4.314 | 0.338 |  |  |  | 15.040 | 0.426 | 42.359 |  |
| ***Analiza(7)_pt4*** | 36.602 | 4.347 |  |  |  |  | 14.200 |  | 44.850 |  |
| ***Analiza(7)_pt5*** | 42.449 | 5.640 | 0.527 | 0.373 |  |  | 13.688 | 0.284 | 37.038 |  |
| ***Analiza(7)_pt6*** | 42.676 | 5.225 | 0.464 | 0.319 |  |  | 14.041 | 0.505 | 36.772 |  |
| ***Analiza(7)_pt7*** | 37.833 | 3.149 | 0.552 |  |  |  | 14.027 | 1.137 | 38.478 | 4.823 |
| ***Analiza(7)_pt8*** | 47.899 | 5.221 | 0.948 | 0.316 |  |  | 13.072 | 0.517 | 32.026 |  |
| ***Analiza(7)_pt9*** | 36.929 | 5.006 | 0.502 |  |  |  | 14.934 | 0.585 | 42.044 |  |
| ***Analiza(7)_pt10*** | 40.471 | 4.050 | 0.223 | 0.339 | 0.000 | 0.305 | 14.271 | 0.342 | 40.000 |  |

Atom %

|  | ***O*** | ***F*** | ***Na*** | ***Mg*** | ***Al*** | ***Si*** | ***P*** | ***S*** | ***Ca*** | ***Ba*** |
| --- | --- | --- | --- | --- | --- | --- | --- | --- | --- | --- |
| ***Analiza(7)_pt1*** | 51.466 | 4.252 | 0.722 | 0.309 |  | 0.303 | 13.544 | 0.371 | 29.034 |  |
| ***Analiza(7)_pt2*** | 52.911 | 5.290 | 0.564 |  |  |  | 12.922 | 0.395 | 27.918 |  |
| ***Analiza(7)_pt3*** | 56.611 | 5.481 | 0.355 |  |  |  | 11.721 | 0.321 | 25.511 |  |
| ***Analiza(7)_pt4*** | 55.880 | 5.589 |  |  |  |  | 11.198 |  | 27.333 |  |
| ***Analiza(7)_pt5*** | 60.807 | 6.804 | 0.526 | 0.352 |  |  | 10.128 | 0.203 | 21.179 |  |
| ***Analiza(7)_pt6*** | 61.147 | 6.305 | 0.462 | 0.300 |  |  | 10.392 | 0.361 | 21.032 |  |
| ***Analiza(7)_pt7*** | 58.560 | 4.105 | 0.595 |  |  |  | 11.216 | 0.879 | 23.775 | 0.870 |
| ***Analiza(7)_pt8*** | 65.653 | 6.026 | 0.904 | 0.285 |  |  | 9.255 | 0.354 | 17.523 |  |
| ***Analiza(7)_pt9*** | 55.714 | 6.360 | 0.527 |  |  |  | 11.638 | 0.440 | 25.320 |  |
| ***Analiza(7)_pt10*** | 59.566 | 5.020 | 0.228 | 0.329 | 0.000 | 0.256 | 10.850 | 0.251 | 23.501 |  |


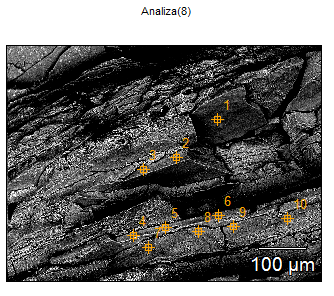


Image Name: layering of the skeleton near the apical end

(GIT 655-2)

Image Resolution: 2048 by 1536

Image Pixel Size: 0.33 µm

Acc. Voltage: 15.0 kV

Magnification: 301

Detector: ULTRADRY


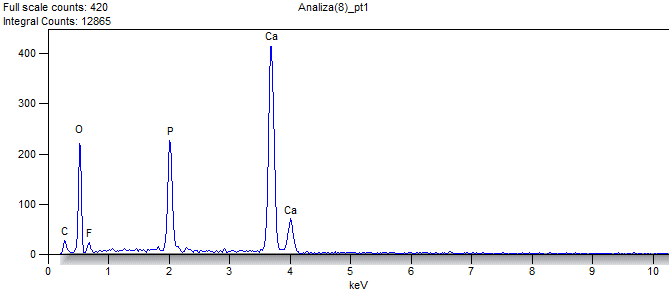


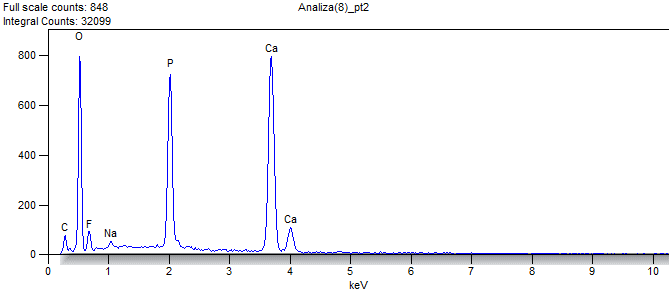


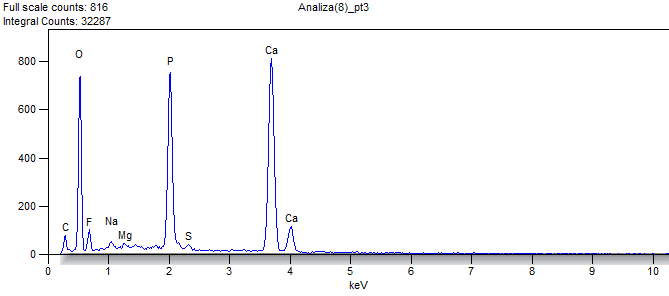


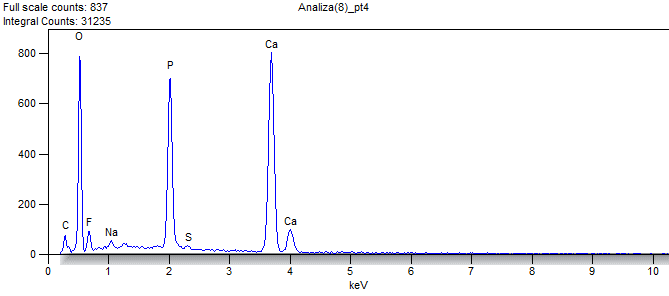


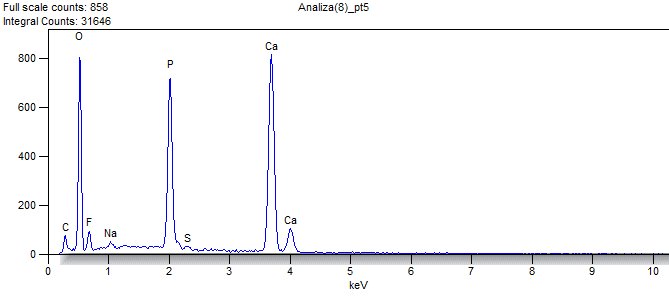


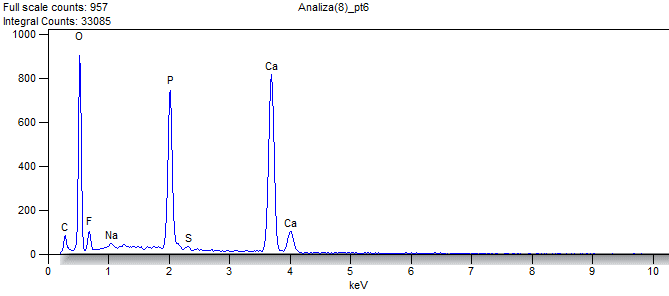


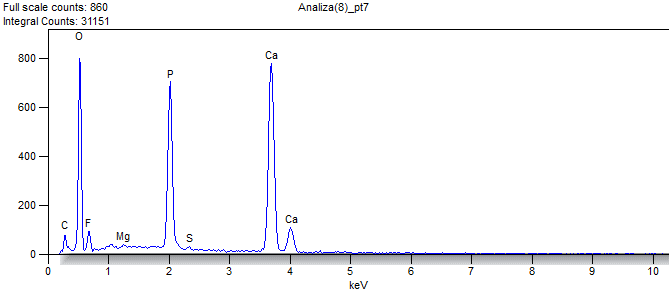


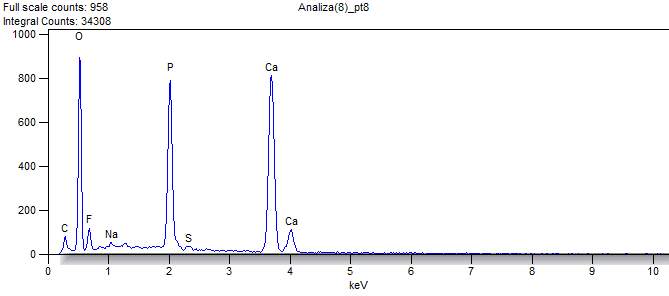


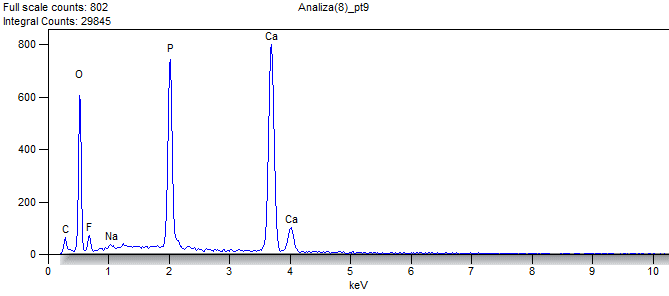


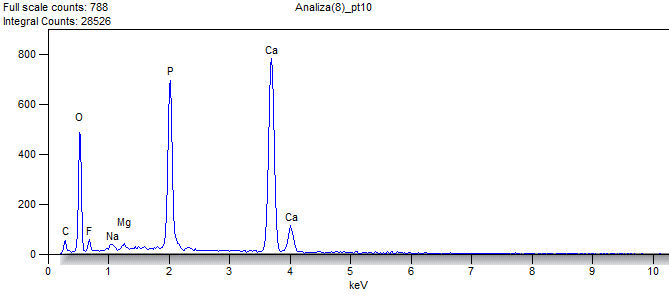


Weight %

|  | ***O*** | ***F*** | ***Na*** | ***Mg*** | ***P*** | ***S*** | ***Ca*** |
| --- | --- | --- | --- | --- | --- | --- | --- |
| ***Analiza(8)_pt1*** | 38.251 | 4.324 |  |  | 11.380 |  | 46.044 |
| ***Analiza(8)_pt2*** | 44.839 | 4.636 | 0.692 |  | 13.982 |  | 35.850 |
| ***Analiza(8)_pt3*** | 42.508 | 5.066 | 0.771 | 0.183 | 14.636 | 0.442 | 36.395 |
| ***Analiza(8)_pt4*** | 44.150 | 5.267 | 0.891 |  | 13.691 | 0.416 | 35.585 |
| ***Analiza(8)_pt5*** | 44.220 | 5.199 | 0.707 |  | 13.800 | 0.317 | 35.756 |
| ***Analiza(8)_pt6*** | 45.435 | 6.162 | 0.559 |  | 13.511 | 0.253 | 34.080 |
| ***Analiza(8)_pt7*** | 44.639 | 5.913 |  | 0.251 | 13.740 | 0.246 | 35.211 |
| ***Analiza(8)_pt8*** | 45.199 | 5.600 | 0.520 |  | 14.085 | 0.306 | 34.291 |
| ***Analiza(8)_pt9*** | 39.644 | 4.170 | 0.503 |  | 15.737 |  | 39.946 |
| ***Analiza(8)_pt10*** | 37.313 | 4.268 | 0.809 | 0.353 | 15.561 |  | 41.697 |

Atom %

|  | ***O*** | ***F*** | ***Na*** | ***Mg*** | ***P*** | ***S*** | ***Ca*** |
| --- | --- | --- | --- | --- | --- | --- | --- |
| ***Analiza(8)_pt1*** | 57.823 | 5.505 |  |  | 8.886 |  | 27.785 |
| ***Analiza(8)_pt2*** | 63.369 | 5.517 | 0.681 |  | 10.207 |  | 20.225 |
| ***Analiza(8)_pt3*** | 60.952 | 6.118 | 0.769 | 0.172 | 10.840 | 0.316 | 20.832 |
| ***Analiza(8)_pt4*** | 62.456 | 6.274 | 0.877 |  | 10.004 | 0.294 | 20.095 |
| ***Analiza(8)_pt5*** | 62.590 | 6.198 | 0.697 |  | 10.090 | 0.224 | 20.203 |
| ***Analiza(8)_pt6*** | 63.348 | 7.235 | 0.542 |  | 9.731 | 0.176 | 18.968 |
| ***Analiza(8)_pt7*** | 62.819 | 7.008 |  | 0.232 | 9.988 | 0.173 | 19.780 |
| ***Analiza(8)_pt8*** | 63.310 | 6.606 | 0.507 |  | 10.191 | 0.214 | 19.173 |
| ***Analiza(8)_pt9*** | 58.661 | 5.197 | 0.518 |  | 12.029 |  | 23.595 |
| ***Analiza(8)_pt10*** | 56.208 | 5.414 | 0.848 | 0.350 | 12.108 |  | 25.073 |


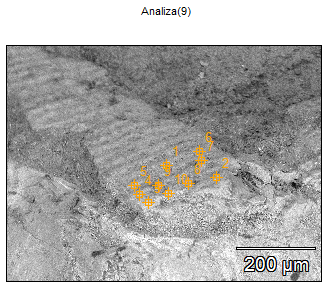


Image Name: annulated sheet (putative periderm)

preserved near the outer margin of a pillar

(GIT 655-1)

Image Resolution: 2048 by 1536

Image Pixel Size: 0.39 µm

Acc. Voltage: 15.0 kV

Magnification: 250

Detector: ULTRADRY


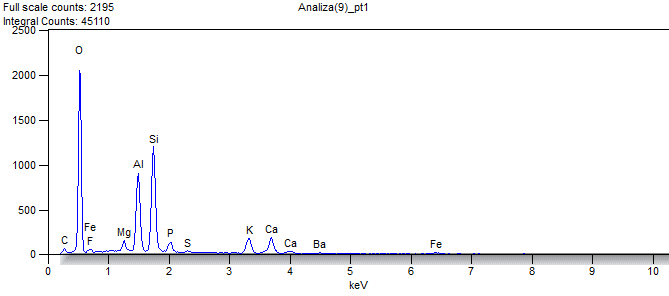


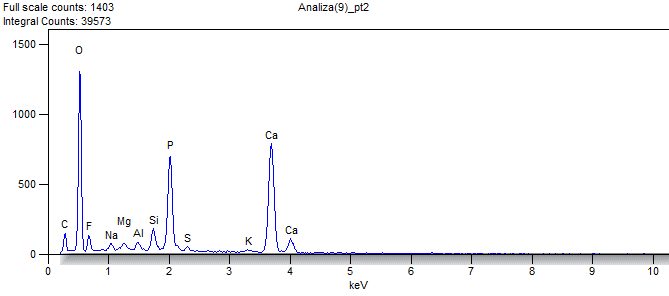


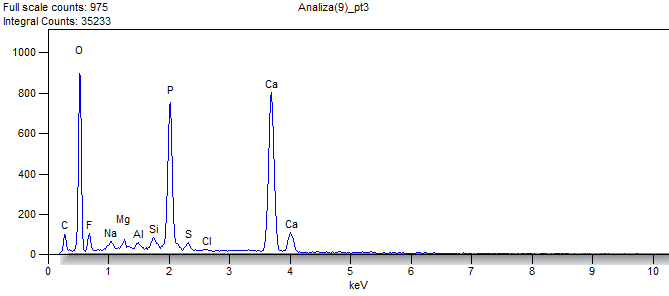


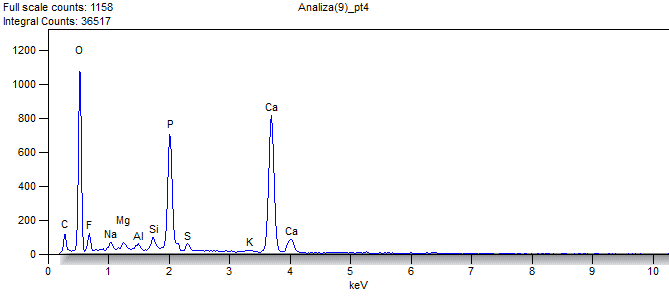


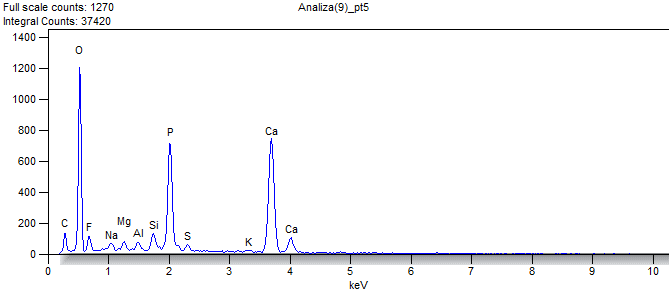


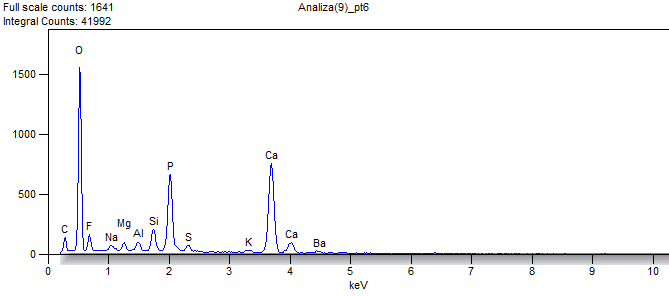


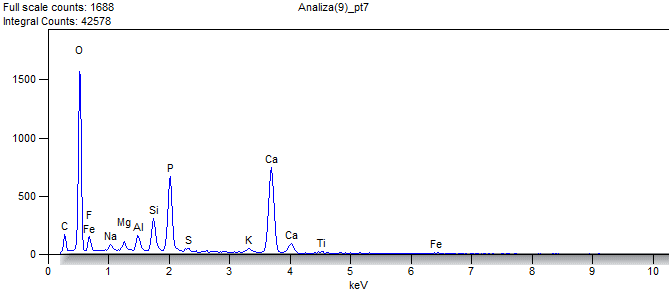


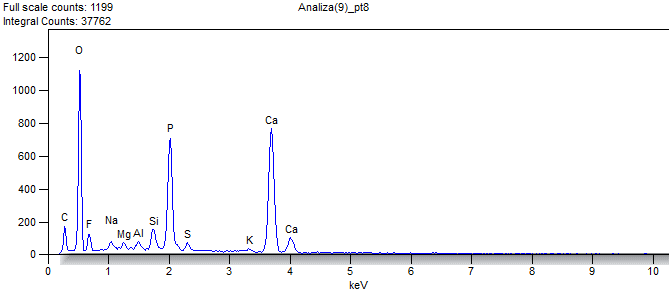


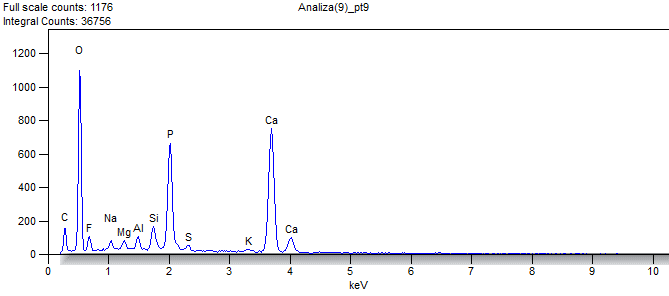


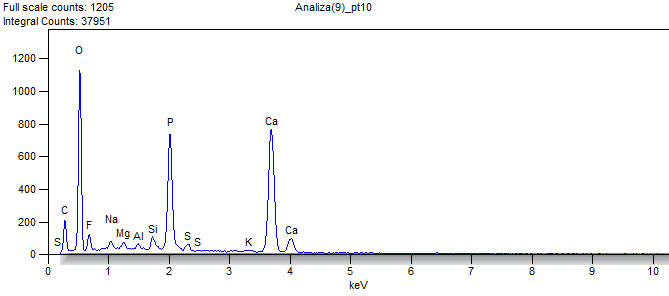


Weight %

|  | ***O*** | ***F*** | ***Na*** | ***Mg*** | ***Al*** | ***Si*** | ***P*** | ***S*** | ***Cl*** | ***K*** | ***Ca*** | ***Ti*** | ***Fe*** | ***Ba*** |
| --- | --- | --- | --- | --- | --- | --- | --- | --- | --- | --- | --- | --- | --- | --- |
| ***Analiza(9)_pt1*** | 54.667 | 0.000 |  | 1.422 | 11.409 | 17.312 | 2.502 | 0.316 |  | 4.587 | 5.018 |  | 1.666 | 1.101 |
| ***Analiza(9)_pt2*** | 51.480 | 6.164 | 0.883 | 0.775 | 0.716 | 2.055 | 10.670 | 0.526 |  | 0.473 | 26.260 |  |  |  |
| ***Analiza(9)_pt3*** | 46.376 | 5.996 | 0.910 | 0.657 | 0.360 | 0.917 | 12.907 | 0.799 | 0.284 |  | 30.795 |  |  |  |
| ***Analiza(9)_pt4*** | 50.424 | 5.107 | 1.009 | 0.674 | 0.371 | 1.121 | 11.638 | 0.740 |  | 0.409 | 28.507 |  |  |  |
| ***Analiza(9)_pt5*** | 49.942 | 7.497 | 0.861 | 0.699 | 0.658 | 1.635 | 11.343 | 0.597 |  | 0.397 | 26.371 |  |  |  |
| ***Analiza(9)_pt6*** | 52.624 | 6.458 | 0.780 | 0.700 | 0.936 | 2.370 | 9.409 | 0.782 |  | 0.557 | 23.323 |  |  | 2.061 |
| ***Analiza(9)_pt7*** | 52.367 | 6.936 | 0.847 | 0.802 | 1.317 | 3.409 | 9.052 | 0.580 |  | 0.900 | 21.890 | 0.650 | 1.250 |  |
| ***Analiza(9)_pt8*** | 49.167 | 6.556 | 1.076 | 0.572 | 0.587 | 1.858 | 11.469 | 0.876 |  | 0.521 | 27.319 |  |  |  |
| ***Analiza(9)_pt9*** | 49.433 | 6.080 | 0.904 | 0.796 | 1.030 | 2.238 | 11.086 | 0.650 |  | 0.516 | 27.267 |  |  |  |
| ***Analiza(9)_pt10*** | 49.732 | 6.228 | 1.027 | 0.656 | 0.343 | 1.113 | 11.647 | 0.772 |  | 0.385 | 28.100 |  |  |  |

Atom %

|  | ***O*** | ***F*** | ***Na*** | ***Mg*** | ***Al*** | ***Si*** | ***P*** | ***S*** | ***Cl*** | ***K*** | ***Ca*** | ***Ti*** | ***Fe*** | ***Ba*** |
| --- | --- | --- | --- | --- | --- | --- | --- | --- | --- | --- | --- | --- | --- | --- |
| ***Analiza(9)_pt1*** | 69.937 | 0.000 |  | 1.198 | 8.655 | 12.616 | 1.654 | 0.201 |  | 2.401 | 2.563 |  | 0.611 | 0.164 |
| ***Analiza(9)_pt2*** | 67.880 | 6.844 | 0.810 | 0.672 | 0.560 | 1.544 | 7.267 | 0.346 |  | 0.255 | 13.822 |  |  |  |
| ***Analiza(9)_pt3*** | 63.779 | 6.944 | 0.871 | 0.595 | 0.294 | 0.718 | 9.169 | 0.549 | 0.176 |  | 16.906 |  |  |  |
| ***Analiza(9)_pt4*** | 67.541 | 5.761 | 0.941 | 0.594 | 0.295 | 0.855 | 8.052 | 0.494 |  | 0.224 | 15.243 |  |  |  |
| ***Analiza(9)_pt5*** | 66.163 | 8.364 | 0.794 | 0.610 | 0.517 | 1.234 | 7.762 | 0.395 |  | 0.215 | 13.946 |  |  |  |
| ***Analiza(9)_pt6*** | 69.242 | 7.156 | 0.714 | 0.606 | 0.731 | 1.776 | 6.395 | 0.514 |  | 0.300 | 12.250 |  |  | 0.316 |
| ***Analiza(9)_pt7*** | 68.279 | 7.616 | 0.769 | 0.688 | 1.018 | 2.532 | 6.097 | 0.377 |  | 0.480 | 11.393 | 0.283 | 0.467 |  |
| ***Analiza(9)_pt8*** | 65.820 | 7.391 | 1.002 | 0.504 | 0.466 | 1.417 | 7.930 | 0.585 |  | 0.285 | 14.599 |  |  |  |
| ***Analiza(9)_pt9*** | 66.142 | 6.851 | 0.842 | 0.701 | 0.817 | 1.706 | 7.662 | 0.434 |  | 0.282 | 14.563 |  |  |  |
| ***Analiza(9)_pt10*** | 66.544 | 7.017 | 0.956 | 0.577 | 0.272 | 0.848 | 8.050 | 0.515 |  | 0.211 | 15.009 |  |  |  |
